# Opposing Effects of Adenosine and Inosine in Human Subcutaneous Fibroblasts May Be Regulated by Third Party ADA Cell Providers

**DOI:** 10.3390/cells9030651

**Published:** 2020-03-07

**Authors:** Carina Herman-de-Sousa, Ana Rita Pinheiro, Diogo Paramos-de-Carvalho, Maria Adelina Costa, Fátima Ferreirinha, Teresa Magalhães-Cardoso, Severino Ribeiro, Julie Pelletier, Jean Sévigny, Paulo Correia-de-Sá

**Affiliations:** 1 Laboratório de Farmacologia e Neurobiologia/MedInUP, Instituto de Ciências Biomédicas Abel Salazar–Universidade do Porto (ICBAS-UP), 4050-313 Porto, Portugal; carina.tsherman@hotmail.com (C.H.-d.-S.); pinheiroar@gmail.com (A.R.P.); diogo.paramos@gmail.com (D.P.-d.-C.); acosta@icbas.up.pt (M.A.C.); mfferreirinha@icbas.up.pt (F.F.); tmcardoso@icbas.up.pt (T.M.-C.); 2 iBiMED—Instituto de Biomedicina, Escola Superior de Saúde, Universidade de Aveiro, 3810-193 Aveiro, Portugal; 3 Departamento de Química, ICBAS-UP, 4050-313 Porto, Portugal; 4 Serviço de Urologia, Centro Hospitalar Universitário do Porto, 4099-001 Porto, Portugal; 0430535801@netcabo.pt; 5 Centre de Recherche du Centre Hospitalier Universitaire de Québec, Université Laval, Québec, QC G1V 4G2, Canada; julie.pelletier@crchudequebec.ulaval.ca (J.P.); jean.sevigny@crchul.ulaval.ca (J.S.); 6 Département de Microbiologie-Infectiologie et d’Immunologie, Faculté de Médecine, Université Laval, Québec, QC G1V 0A6, Canada

**Keywords:** human subcutaneous fibroblasts, inosine, adenosine A_2A_ receptor, adenosine A_3_ receptor, exchange protein activated by cyclic AMP (EPAC) pathway, cells proliferation, collagen production

## Abstract

Human subcutaneous fibroblasts (HSCF) challenged with inflammatory mediators release huge amounts of ATP, which rapidly generates adenosine. Given the nucleoside’s putative relevance in wound healing, dermal fibrosis, and myofascial pain, we investigated the role of its precursor, AMP, and of its metabolite, inosine, in HSCF cells growth and collagen production. AMP (30 µM) was rapidly (t½ 3 ± 1 min) dephosphorylated into adenosine by CD73/ecto-5′-nucleotidase. Adenosine accumulation (t½ 158 ± 17 min) in the extracellular fluid reflected very low cellular adenosine deaminase (ADA) activity. HSCF stained positively against A_2A_ and A_3_ receptors but were A_1_ and A_2B_ negative. AMP and the A_2A_ receptor agonist, CGS21680C, increased collagen production without affecting cells growth. The A_2A_ receptor antagonist, SCH442416, prevented the effects of AMP and CGS21680C. Inosine and the A_3_ receptor agonist, 2Cl-IB-MECA, decreased HSCF growth and collagen production in a MRS1191-sensitive manner, implicating the A_3_ receptor in the anti-proliferative action of inosine. Incubation with ADA reproduced the inosine effect. In conclusion, adenosine originated from extracellular ATP hydrolysis favors normal collagen production by HSCF via A_2A_ receptors. Inhibition of unpredicted inosine formation by third party ADA cell providers (e.g., inflammatory cells) may be a novel therapeutic target to prevent inappropriate dermal remodeling via A_3_ receptors activation.

## 1. Introduction

Subcutaneous connective tissue disorganization is a common feature in patients with chronic pain, possibly as a result of tissue inflammation and fibrosis [1,2,3]. Chronic deformation of the myofascial system such as increased deposition of type I collagen causes mechanoreceptors to become nociceptors and may develop chronic pain. Fibroblasts represent the base of the fascial system, a connective tissue structure that covers and affects every area of the body [4]. These cells play a key role in transmitting nociceptive information, which is useful for proper functioning of the body system.

Data from previous studies indicate that purines may potentially play a role in subcutaneous inflammation, nociception, and connective tissue remodeling, namely due to the activation of nucleotide-sensitive P2 purinoceptor subtypes [5,6,7,8]. However, little information is available regarding the expression and the function of P1 purinoceptor subtypes in the human subcutaneous connective tissue (see, e.g., [9]; reviewed in [10]). These receptors are specifically activated by adenosine, which is the main metabolite of the ectonucleotidase cascade. Our group demonstrated that extracellular ATP and ADP are broken down by membrane-bound NTPDase1 (also called CD39 or apyrase, EC 3.6.1.5) and NTPDase2 (CD39L1, EC 3.6.1.3) into AMP at the surface of human subcutaneous fibroblasts (HSCF), which may be then promptly dephosphorylated into adenosine by ecto-5′-nucleotidase (also called CD73, EC 3.1.3.5) [7,8]. Alternatively, adenosine can be released as such from most cell types via equilibrative nucleoside transporters [11]. The extracellular adenosine accumulation is limited (30–300 nM range) by cellular reuptake and/or deamination into inosine by adenosine deaminase (ADA, EC 3.5.44) [11].

Mounting evidence indicates that extracellular adenosine levels increase dramatically in tissues submitted to stressful conditions, such as ischemia, hypoxia, and inflammation. The nucleoside is a potent endogenous modulator of cardiovascular function, neuronal activation, inflammation, and connective tissue repair. However, excessive extracellular adenosine accumulation in the dermis (and in many other locations) may lead to scar progression and fibrosis [12]. Thus, increasing adenosine production in response to stressful stimuli might have a dual modulatory role in tissue homeostasis [13]. While adenosine first acts as an alarm molecule reporting injury to surrounding cells in order to trigger protective responses, persistent accumulation of adenosine may lead to maladaptive organ responses, as observed in chronic inflammation, fibrosis, and/or delayed tissue healing.

Adenosine effects are mediated by differential activation of four G protein-coupled P1 purinoceptors, namely A_1_, A_2A_, A_2B,_ and A_3_; each one may undertake a specific role [14,15]. Depending on the tissue, evidence shows that activation of the same receptor subtype may cause opposing effects. For instance, the adenosine A_2B_ receptor inhibits fibrosis in the heart while promoting fibrosis of the lung [16]. In the skin, the liver, and the lungs, both A_2A_ and A_2B_ receptors favor fibrosis (reviewed in [12]). Nevertheless, the dynamics of adenosine receptor expression and activation in inflammatory conditions and tissue remodeling/fibrosis are still controversial [17]. The putative therapeutic impact of A_1_ and A_2A_ receptor agonists in myofascial pain is challenged by significant cardiovascular side effects [18]. On the other hand, identification of novel, powerful, and selective A_3_ receptor ligands opens new avenues for elucidation of their therapeutic potential in chronic myofascial conditions [14], but this is weakened by scarcity of studies about their expression and function in fibroblasts of the human subcutaneous tissue [19,20].

Taking into consideration (1) that controversy still exists regarding the participation of adenosine in wound healing, dermal fibrosis, and myofascial pain, and (2) that the nucleoside is promptly generated from the extracellular catabolism of released adenine nucleotides by human fibroblasts challenged with inflammatory mediators (e.g., histamine, bradykinin), we decided to investigate the long-term role of the adenosine precursor, AMP, and its deamination metabolite, inosine, in the proliferation and the collagen production by fibroblasts of the human subcutaneous tissue.

## 2. Experimental Procedures

### 2.1. Cell Cultures

Human fibroblasts were isolated from the subcutaneous tissue of male organ donors (45 ± 6 years old, n = 36) with no clinical history of connective tissue disorders. All subjects gave their informed consent for inclusion before they participated in the study. The protocol was approved by the Ethics Committee of Hospital Geral de Santo António SA (University Hospital) and of Instituto de Ciências Biomédicas de Abel Salazar (Medical School) of University of Porto. The investigation conforms to the principles outlined in the Declaration of Helsinki.

Subcutaneous tissues were maintained at 4–6 °C in M-400 transplantation solution (4.190 g/100 mL mannitol, 0.205 g/100 mL KH_2_PO_4_, 0.970 g/100 mL K_2_HPO_4_·3H_2_O, 0.112 g/100 mL KCl, and 0.084 g/100 mL NaHCO_3_, pH 7.4) until used, which was between 2 and 16 h after being harvested [21]. Cells were then obtained by the explant technique and cultured in Dulbecco’s Modified Eagle’s Medium (DMEM) medium supplemented with 10% fetal bovine serum (FBS), 2.5 µg/mL of amphotericin B, and 100 U/mL of penicillin/streptomycin, at 37 °C in a humidified atmosphere of 95% air and 5% CO_2_. Medium was replaced twice a week. Primary cultures were maintained until near confluence (~3–4 weeks), then adherent cells were enzymatically released with 0.04% trypsin-EDTA solution plus 0.025% type I collagenase in phosphate-buffered saline (PBS). The resultant cell suspension was plated and maintained in the same conditions mentioned above. All the experiments were performed in the first subculture.

### 2.2. Human CD73 Antibody Production

Antibodies to human CD73 were generated by cDNA immunization in Hartley guinea pigs and were validated (http://ectonucleotidases-ab.com/documents/human5-nucleotidase_GP.pdf).

### 2.3. Immunocytochemistry

Human fibroblasts were seeded in chamber slides at a density of 2.5 × 10^3^ cells/mL and allowed to grow for 7 and 28 days. Cultured cells were fixed in 4% paraformaldehyde (PFA) in PBS for 10 min, washed 3 times in PBS (10 min each), and subsequently incubated with blocking buffer I (10% FBS, 1% bovine serum albumin (BSA), and 0.1% Triton X, 0.05% NaN_3_) for 1 h. Primary antibodies diluted in blocking buffer II (5% FBS, 1% BSA, 0.1% Triton X, 0.05% NaN_3_) were applied [mouse anti-porcine vimentin (1:75, DAKO); rabbit anti-human collagen I (1:50, AbDSerotec, Kidlington, UK); rabbit anti-rat A_1_ 1:50, and rabbit anti-human A_2B_ (1:75, Chemicon, Temecula, CA, USA); rabbit anti-canine A_2A_ (1:100, Alpha Diagnostic, San Antonio, TX, USA); rabbit anti-human A_3_ (1:150, Alomone, Jerusalem, Israel); and guinea-pig anti-human CD73 h5′NT-2_C_(I4) (1:300, available at http://ectonucleotidases-ab.com)], and the slides were incubated overnight at 4 °C. After incubation, cells were washed 3 times in PBS 1× (10 min each). The donkey anti-rabbit IgG Alexa Fluor 488, the donkey anti-mouse IgG Alexa Fluor 568, the donkey anti-guinea pig IgG Alexa Fluor 568, and the donkey anti-goat IgG Alexa Fluor 633 secondary antibodies (Molecular Probes, Eugene, OR, USA) were diluted in blocking buffer II (5% FBS, 1% BSA, 0.1% Triton X) and applied for 1 h protected from light. A last wash was performed with PBS 1X, and glass slides were mounted with VectaShield medium and stored at 4 °C. Negative controls were carried out by replacing the primary antibodies with non-immune serum; cross-reactivity for the secondary antibodies was tested in control experiments in which primary antibodies were omitted (Supplementary Figure S1). Observations were performed and analyzed with an Olympus FV1000 confocal microscope (Olympus FV1000, Tokyo, Japan) [7,8,22].

### 2.4. SDS-PAGE and Western Blotting

Human subcutaneous fibroblasts (HSCF) were seeded in chamber slides at a density of 6.0 × 10^3^ cells/mL and allowed to grow for 7 and 28 days in culture. Cells were homogenized in a lysis buffer with the following composition: 50 mM Tris-HCl (pH 8.0), 150 mM NaCl, 0.5% sodium deoxycholate, 1% Triton-X-100, 0.1% SDS, and a protease inhibitor cocktail. Protein content of the samples was evaluated using the Pierce BCA Protein Assay kit according to the manufacturer’s instructions (Thermo Fisher Scientific, Waltham, MA, USA). Samples were solubilized in SDS reducing buffer (0.125 mM Tris-HCl, 4% SDS, 0.004% bromphenol blue, 20% glycerol, and 10% 2-mercaptoethanol, pH 6.8 at 70 °C for 10 min), subjected to electrophoresis in 10% SDS-polyacrylamide gels, and electrotransferred onto PVDF membranes (MilliPore, Burlington, MA, USA). Protein loads were 150 μg for A_2A_R, A_3_R, and CD73. The membranes were blocked for 1 h in Tris buffered saline (TBS: 10 mM Tris-HCl, pH 7.5, 150 mM NaCl) containing 0.05% Tween 20 + 5% BSA. Membranes were subsequently incubated with rabbit anti-human A_3_R (1:200, Alomone, Jerusalem, Israel), rabbit anti-human A_2A_R (1:200, Alpha Diagnostics, San Antonio, TX, USA), and rabbit anti-CD73 (1:500; h5′NT-2_L_ I5) available at http://ectonucleotidases-ab.com) in the above blocking buffer overnight at 4 °C. Membranes were washed three times for 10 min in 0.1% Tween 20 in TBS and then incubated with donkey anti-rabbit IgG (HRP) 1:70000 (Abcam Plc, Cambridge, UK) secondary antibody for 60 min at room temperature. For normalization purpose, membranes were incubated with mouse anti-glyceraldehyde 3-phosphate dehydrogenase (GAPDH) (1:200; 37 kDa Santa Cruz Biotechnology, Dallas, TX, USA), rabbit anti-β-actin (1:5000; 42 kDa; Abcam Plc, Cambridge, UK), and rabbit anti-β-tubulin (1:2500; 50 kDa; Abcam Plc, Cambridge, UK) antibodies following the procedures described above. The antigen-antibody complexes were visualized using the ChemiDoc MP imaging system (Bio-Rad Laboratories, Hercules, CA, USA). Gel band image densities were quantified with ImageJ (National Institute of Health, Bethesda, MD, USA).

### 2.5. Enzymatic Kinetic Experiments and HPLC Analysis

After a 30 min equilibration period at 37 °C, HSCF grown for 11 days were incubated with 3 or 30 μM of AMP or adenosine, which was added to the culture medium at zero time. Samples (75 μL) were collected from each well at different times up to 30 min for high-performance liquid chromatography (HPLC, LaChrome Elite, Merck, Germany) analysis of the variation of substrate disappearance and product formation [7,8,22]. Adenosine catabolism analysis was performed at room temperature with a LiChrospher 100 RP-18 (5 µm) column (Merck) by isocratic reverse-phase HPLC-UV set at 254 nm. The eluent was composed of 91% of 100 mM KH2PO4, pH = 7, and 9% of methanol during 10 min with a constant rate flow of 1.25 mL/min. Under these conditions, the retention times of adenosine and its metabolites were as follows: adenosine (8.03 min), inosine (2.73 min) and hypoxantine (1.78 min). For the AMP catabolism analysis, we used a linear gradient (100% 100 mM KH_2_PO_4_, pH 7, to 100% 100 mM KH_2_PO_4_, pH 7, 30% methanol) for 10 min with a constant rate flow of 1.25 mL/min. Under these conditions, the retention times of metabolites were as follows: AMP (2.17 min), hypoxanthine (3.07 min), inosine (5.09 min), and adenosine (7.51 min).

Concentrations of the substrate and the products were plotted as a function of time (progress curves). The following parameters were analyzed for each progress curve: half-life time (t½) of the initial substrate, time of appearance of the different concentrations of the products, concentration of the substrate, or any product remaining at the end of the experiment. The spontaneous degradation of AMP and adenosine at 37 °C in the absence of the cells was negligible (0–3%) over 30 min. At the end of the experiments, the remaining incubation medium was collected and used to quantify the lactate dehydrogenase (LDH, EC 1.1.1.27) activity. The negligible activity of LDH in the samples collected at the end of the experiments is an indication of the integrity of the cells during the experimental period.

### 2.6. Cell Viability/Proliferation

Viability/proliferation studies included the 3-[4,5-dimethylthiazol-2-yl]-2,5-diphenyltetrazolium bromide (MTT) assay as previously described [7,8]. HSCF were seeded in flat bottom 96 well plates at a density of 3 × 10^4^ cells/mL and cultured in supplemented DMEM. Cell cultures were routinely monitored by phase contrast microscopy and characterized at days 1, 7, 14, 21, and 28. The MTT assay consists of the reduction of MTT to a purple formazan reaction product by viable cells. In the last 4 h of each test period, cells were incubated with 0.5 mg/mL of MTT for 4 h in the conditions referred above. The medium was carefully removed, decanted, and the stained product dissolved with DMSO before absorbance (A) determination at 600 nm using a microplate reader spectrometer (Synergy HT, BioTek, Vermont, VT, USA). Results were expressed as A/well.

### 2.7. Total Collagen Determination

Collagen determination was performed using the Sirius Red colorimetric assay that stains equally well collagen types I and III [23], which are the two main collagen types existing in the human skin, normally at a ratio of 4:1 [24]. HSCF were cultured as described for the viability/proliferation studies. Cell layers were washed twice in PBS before fixation with Bouin’s fluid for 1 h. The fixation fluid was removed by suction, and the culture plates were washed by immersion in running tap water for 15 min. Culture dishes were allowed to air dry before adding the Sirius Red dye (Direct Red 80). Cells were stained for 1 h under mild shaking on a microplate shaker. To remove non-bound dye, stained cells were washed with 0.01 N hydrochloric acid and then dissolved in 0.1 N sodium hydroxide for 30 min at room temperature using a microplate shaker. Optical density was measured at 550 nm against 0.1 N sodium hydroxide as blank. Results were expressed as A/well.

### 2.8. Materials and Reagents

Amphotericin B, bovine serum albumin (BSA), Dulbecco’s Modified Eagle’s Medium (DMEM), ethylene diaminetetraacetic acid (EDTA), fetal bovine serum (FBS), penicillin/streptomycin, phosphate buffered saline system (PBS), and type I collagenase, as well as 4-[2-[[6-Amino-9-(N-ethyl-β-D-ribofu-ranuronamidosyl)-9H-purin-2-yl]amino]ethyl] benzene-propanoic acid hydrochloride (CGS 21680), inosine (hypoxanthine 9-β-D-ribofuranoside), adenosine 5′-monophosphate (AMP) sodium salt, adenosine deaminase (ADA type X from bovine spleen, buffered aqueous glycerol solution), Concanavalin A (type IV from *Canavalia ensiformis*, Jack bean), dipyridamole, 9-(tetrahydro-2-furanyl)-9H-purin-6-amine,9-THF-Ade (SQ 22,536), N-[2-(p-bromocinnamylamino)ethyl]-5-isoquinolinesulfonamide dihydrochloride (H-89) dihydrochloride hydrate, α-[2-(3-chlorophenyl) hydrazinylidene]-5-(1,1-dimethylethyl)-b-oxo-3 isoxazolepropanenitrile (ESI-09), and 3-ethyl-5-benzyl-2-methyl-4-phenylethynyl-6-phenyl-1,4-(±)-dihydropyridine-3,5-dicarboxylate (MRS 1191) were purchased from Sigma-Aldrich. The 2-(2-Furanyl)-7-[3-(4-methoxyphenyl-)propyl]-7H-pyrazolo[4,3-e][1,2,4]triazolo[1,5-c]p-yrimidin-5-amine (SCH 442416) and 1-[2-chloro-6-[[(3-iodophenyl)methyl]amino]-9H-purin-9-yl]-1-deoxy-N-methyl-β-D-ribofuranuronamide (2Cl-IB-MECA) were obtained from Tocris Cookson Inc, Dimethylsulphoxide (DMSO) and glacial acetic acid were obtained from Merck and Bouin’s solution was acquired from Panreac. CGS 21680, 2Cl-IB-MECA, Dipyridamole, MRS119, ESI-09, H-89, and SCH 442416 were prepared in dimethyl sulfoxide (DMSO). All other drugs were prepared in distilled water or culture medium. All stock solutions were stored as frozen aliquots at −20 °C. Dilutions of these stock solutions were made daily, and appropriate solvent controls were done. No statistically significant differences between control experiments made in the absence or in the presence of DMSO at the maximal concentration used (0.05% *v*/*v*) were observed. The pH of the solutions did not change by the addition of the drugs in the maximum concentrations applied to the preparations. The 96-well tissue culture plates were purchased from Corning; chamber slides were from Nunc.

### 2.9. Presentation of Data and Statistical Analysis

Data are expressed as mean ± SD from an n number of experiments/cells/individuals. Statistical analysis was carried out using Graph Pad Prism 8.3.0 software (La Jolla, CA, USA). One- or two-way ANOVA followed by the Tukey’s multicomparison test was used only if F was significant and there was no variance inhomogeneity; few outliers were identified and removed using the ROUT method with a Q = 1%. *p* < 0.05 (two-tailed) values were considered statistically significant.

## 3. Results

### 3.1. Human Subcutaneous Fibroblasts Express Ecto-5′-Nucleotidase/CD73 and Adenosine A_2A_ and A_3_ Receptor Subtypes

**Figure 1:**
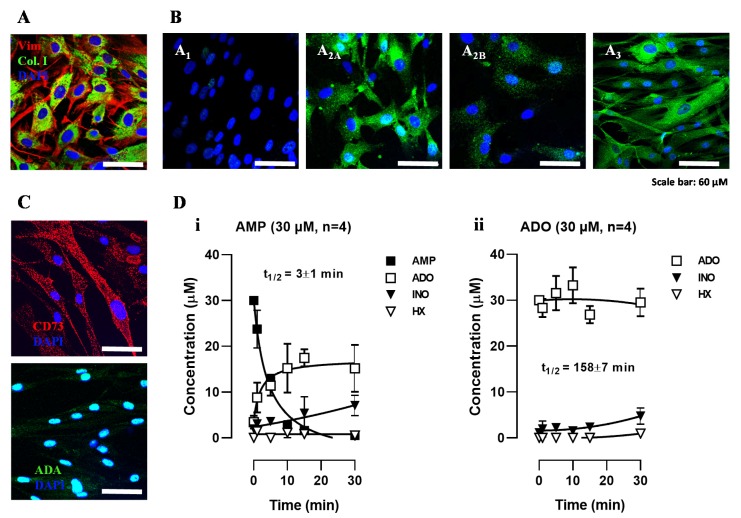
Human subcutaneous fibroblasts (HSCF) express ecto-5′-nucleotidase/CD73, the enzyme responsible for AMP dephosphorylation into adenosine, but lack adenosine deaminase (ADA), resulting in extracellular adenosine accumulation that may signal via co-expressed A_2A_ and A_3_ receptor subtypes. Panel **A** shows the immunoreactivity against fibroblast cell markers, vimentin (red), and type I collagen (green). The panel **B** shows that HSCF stain positively against A_2A_ and A_3_ receptors (green), with very little amounts of A_2B_ and A_1_ receptor subtypes. The panel **C** shows that HSCF are ecto-5′-nucleotidase/CD73 positive ADA negative cells. Nuclei are stained in blue with DAPI; scale bar is 60 μm. Micrographs were obtained from at least three different individuals with a laser scanning confocal microscope using the same acquisition settings. Panel **D** shows the time course of the extracellular catabolism of AMP (30 µM, (**i**) and adenosine (ADO, 30 μM, (**ii**) in HSCF cultures allowed to grow for 11 days. AMP and ADO were added to the culture medium at time zero. Samples (75 μL) were collected from each well at indicated times in the abscissa. Each collected sample was analyzed by HPLC to separate and quantify AMP (filled squares), adenosine (open squares), inosine (filled triangles), and hypoxanthine (open triangles). Each point represents pooled data from four individuals; two replicas were performed in each individual experiment. Vertical bars represent SEM and are shown when they exceed the symbols in size. The calculated half-life time (t½, min) for each initial substrate is shown for comparison.

Human subcutaneous fibroblasts (HSCF) are elongated cells with a characteristic spindle-shaped morphology exhibiting positive immunoreactivity against fibroblast-cell markers, such as vimentin and type I collagen (Figure 1A; see also [7,8]). From the four adenosine receptor subtypes, cultured HSCF showed strong immunoreactivity against A_2A_ and A_3_ receptor subtypes, with faint A_2B_ receptor staining and no evidence of the A_1_ receptor being present in these cells (Figure 1B). The low immunoreactivity against A_1_ and A_2B_ receptors could not be attributed to deficient quality of the antibodies, because positive identification of the two receptors was previously demonstrated by our group in human primary bone marrow stromal cells undergoing osteogenic differentiation using the same experimental procedure and antibodies (anti-A1 #AB1587P and anti-A2B #AB1589P from Chemicon, Temecula, CA, USA) (Supplementary Figure S1; see also [22]).

Figure 1C shows that cultured HSCF expressed significant amounts of ecto-5′-nucleotidase/CD73, the enzyme that is responsible for extracellular AMP dephosphorylation enabling adenosine formation from released adenine nucleotides. The lack of adenosine deaminase (ADA) staining at the plasma membrane of these cells (Figure 1C) suggests that adenosine may accumulate in the extracellular milieu strengthening activation of plasma membrane-bound adenosine receptors.

### 3.2. Adenosine Formation from AMP Overcomes its Deamination into Inosine Favoring Accumulation of the Nucleoside in HSCF Cultures

Figure 1D illustrates the time course of the extracellular catabolism of AMP and adenosine in cultured HSCF. AMP (30 µM) was rapidly (t½ 3 ± 1 min, n = 4) dephosphorylated into adenosine by ecto-5′-nucleotidase/CD73; little amounts of inosine were detected 15 min after application of the nucleotide. The progress curve of adenosine (30 µM) disappearance shows that the nucleoside was slowly (t½ 158 ± 17 min, n = 4) deaminated into inosine with almost no formation of hypoxanthine in HSCF cultures. Inosine reached a maximal concentration of 4 ± 3 µM 30 min after adenosine (30 µM) application. The absence of AMP formation from adenosine suggests that no extracellular adenosine kinase (ADK, E.C. 2.7.1.20) activity was present in HSCF. The stoichiometry of extracellular adenosine disappearance and metabolites formation was kept fairly constant throughout the reaction time period, i.e., the sum of the initial substrate, the adenosine, plus its metabolites was roughly 30 µM in all considered time points, leading to the conclusion that cellular adenosine uptake was irrelevant under the present experimental conditions.

Using a smaller (3 µM) concentration, adenosine was metabolized with a half-life time (t½) of 40 ± 8 min (n = 4), leading to a maximal inosine concentration of 1 ± 1 µM 30 min after adenosine application (data not shown). The semi-logarithmic representation of progress curves obtained by polynomial fitting of the nucleoside catabolism show a linear pattern (y = −0.0051x + 0.4578 and R^2^ = 0.99 for 3 µM: y = −0.0015x + 1.4640 and R² = 0.99 for 30 µM). Given that the slope of the progress curves decreased upon increasing the concentration of the substrate from 3 to 30 µM, data indicate that the kinetics of the catabolism of adenosine was slower upon increasing the concentration of the nucleoside.

Enzymatic kinetic studies confirm our prediction that HSCF express small amounts of ADA, which is rapidly overcome by adenosine formation from released adenine nucleotides, thus favoring adenosine accumulation with little inosine production in the extracellular milieu.

### 3.3. AMP Favors Collagen Production via Adenosine A_2A_ Receptors Activation Coupled to the Adenylyl Cyclase (AC)/Exchange Protein Activated by Cyclic AMP (EPAC) Pathway in HSCF

**Figure 2:**
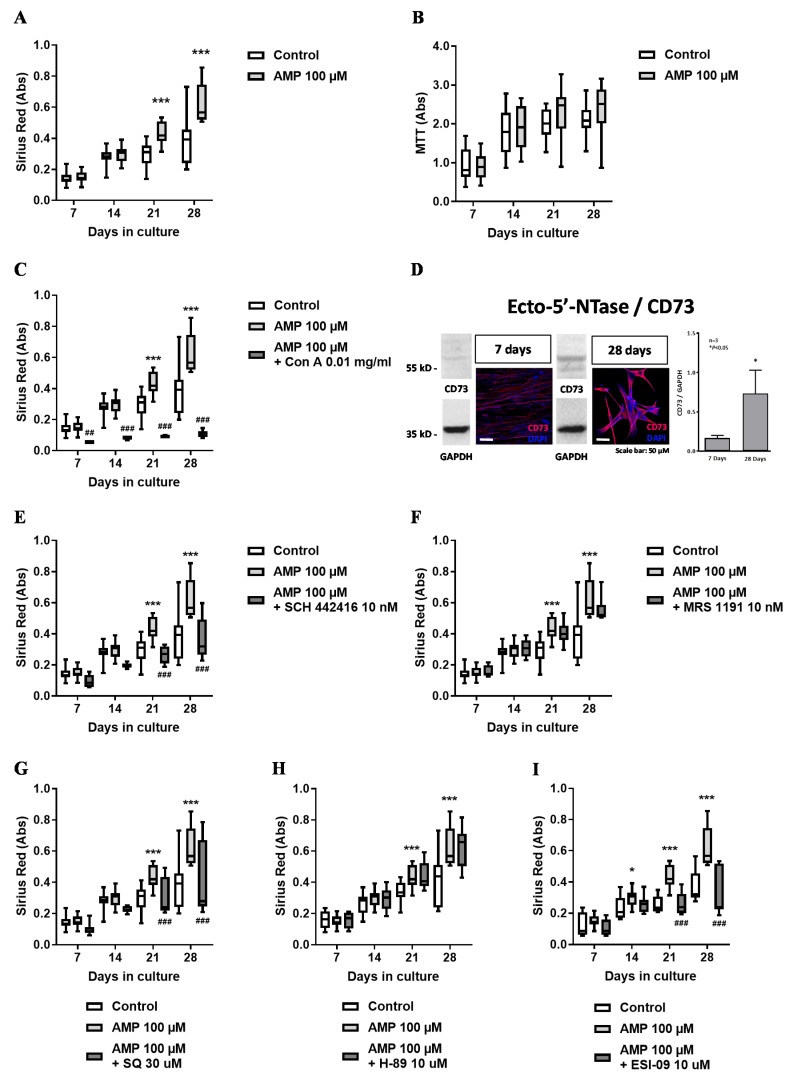
Preferential effect of the adenosine precursor, AMP (100 µM), on collagen production (Sirius Red staining, panel **A**) by human subcutaneous fibroblasts (HSCF) kept in culture for 28 days with no significant effect on cells proliferation/viability (3-[4,5-dimethylthiazol-2-yl]-2,5-diphenyltetrazolium bromide (MTT) assay, panel **B**). The effect of AMP (100 µM) was prevented by the ecto-5′-nucleotidase/CD73 inhibitor, concanavalin A (0.01 mg/mL, panel **C**), indicating that the nucleotide must be converted into adenosine to exert its pro-fibrotic effect. Panel **D** shows that ecto-5′-nucleotidase/CD73 immunoreactivity increases with the time of the cells in culture, as evidenced both by immunofluorescence confocal microscopy and by Western blot analysis normalized by the glyceraldehyde 3-phosphate dehydrogenase (GAPDH) content of the cells. Nuclei are stained in blue with DAPI. Shown are representative data from three different individuals; experiments were performed in triplicate. The involvement of A_2A_ receptors coupled to the adenylate cyclase (AC)/exchange protein activated by cyclic AMP (EPAC) pathway was proven by prevention of the pro-fibrotic effect of AMP with SCH442416 (10 nM, panel **E**), SQ22536 (30 µM, panel **G**), and ESI-09 (10 µM, panel **I**), which blocked the A_2A_ receptor, AC and EPAC, respectively. Blockage of the A_3_ receptor and of protein kinase A with MRS1191 (10 nM, panel **F**) and H-89 (10 µM, panel **H**), respectively, was devoid of effect. Boxes and whiskers represent pooled data from at least three different individuals; 4–6 replicas were performed for each individual. * *p* < 0.05 and *** *p* < 0.001 (two-way ANOVA) represent significant differences compared to control conditions in the same individual; ^##^ *p* < 0.01 and ^###^ *p* <0.001 (two-way ANOVA) represent significant differences compared to the effect of AMP in the same set of experiments.

Incubation of HSCF cultures with AMP (30–100 µM) for 28 days concentration-dependently increased (*p* < 0.05) collagen production (Sirius Red assay; Figure 2A) without affecting cells growth (MTT assay; Figure 2B). The pro-fibrotic effect of AMP (100 µM) was fully prevented by the non-nucleotide inhibitor of ecto-5′-nucleotidase/CD73, concanavalin A (0.01 mg/mL) (Figure 2C). Interestingly, the facilitatory effect of AMP on collagen production was more evident at culture days 21 and 28. These time points coincided with increases in the ecto-5′-nucleotidase/CD73 protein content shown by immunocytochemistry and Western blot analysis comparing culture days seven and 28 (Figure 2D).

Results suggest that promotion of collagen production induced by AMP requires progressive extracellular adenosine formation by ecto-5′-nucleotidase/CD73 in mature HSCF. The involvement of co-expressed adenosine receptors on AMP-induced collagen production by HSCF was investigated using subtype-selective A_2A_ and A_3_ receptor antagonists, SCH442416 (10 nM) and MRS1191 (10 nM), respectively. The facilitatory effect of AMP (100 µM) on collagen production was prevented upon blocking the A_2A_ receptor with SCH442416 (10 nM) (Figure 2E), while selective blockage of the A_3_ receptor with MRS1191 (10 nM) was virtually ineffective (Figure 2F). Data suggest that AMP favors collagen production by HSCF via adenosine A_2A_ receptors activation.

Next, we set to investigate the intracellular signaling pathway coupling adenosine A_2A_ receptors activation to collagen production by HSCF in the presence of the adenosine precursor, AMP. Most commonly, the A_2A_ receptor couples to the adenylyl cyclase (AC)/cyclic AMP pathway. Selective inhibition of AC with SQ22536 (30 µM; Figure 2G) as well as inhibition of the exchange protein activated by cyclic AMP (EPAC) with ESI-09 (10 µM; Figure 2I), but not of the protein kinase A with H-89 (10 µM; Figure 2H), prevented AMP-induced facilitation of collagen production by HSCF. Blockage of cyclic AMP specific type IV phosphodiesterase with rolipram (300 µM) mimicked the pro-fibrotic effect of AMP (100 µM) on collagen production at culture days 21 and 28 without affecting HSCF cells growth (data not shown). Overall, data suggest that adenosine A_2A_ receptors coupled to the AC/EPAC pathway are involved in the pro-fibrotic effect of AMP in cultured HSCF.

### 3.4. Effect of Adenosine A_2A_ and A_3_ Receptor Agonists on Proliferation/Viability and Collagen Production by HSCF

**Figure 3:**
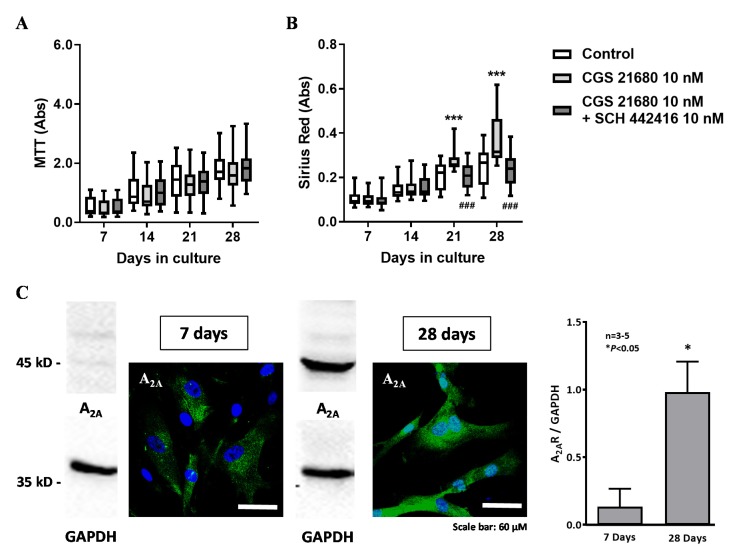
The adenosine A_2A_ receptor agonist, CGS21680 (10 nM), mimicked the effect of AMP on collagen production (Sirius Red staining, panel **B**) while having no effect on human subcutaneous fibroblasts (HSCF) growth (MTT assay, panel **A**). The pro-fibrotic effect of CGS21680 (10 nM) was abrogated by the selective A_2A_ receptor antagonist, SCH442416 (10 nM). Boxes and whiskers represent pooled data from at least three different individuals; 4–6 replicas were performed for each individual. *** *p* < 0.001 (two-way ANOVA) represent significant differences compared to control conditions; ^###^ *p* < 0.001 (two-way ANOVA) represent significant differences compared to the effect of CGS21680 in the same set of experiments. Panel **C** shows that A_2A_ receptor expression (green) increases from day seven to 28, as evidenced both by immunofluorescence confocal microscopy and by Western blot analysis normalized by the GAPDH content of the cells. Nuclei are stained in blue with DAPI. Shown are representative data from three (7 days) to five (28 days) different individuals; experiments were performed in triplicate. * *p* < 0.05 (Student’s *t*-test) represent significant differences compared to culture day seven.

The facilitatory effect of AMP (100 µM) on collagen production was mimicked by the selective A_2A_ receptor agonist CGS21680C (10 nM) (Figure 3B). Likewise, the A_2A_ receptor agonist also did not modify cell viability/proliferation of HSCF (Figure 3A). The facilitatory effect of CGS 21680 (10 nM) on collagen production by HSCF was prevented by co-application of the selective A_2A_ receptor antagonist, SCH442416 (10 nM) (Figure 3B); neither CGS 21680 (10 nM) alone or in the presence of SCH442416 (10 nM) affected viability/proliferation of HSCF (Figure 3A). Like that observed with AMP (100 µM), the pro-fibrotic effect of CGS 21680 (10 nM) was more evident at culture days 21 and 28, when HSCF exhibited the highest A_2A_ receptor immunoreactivity, as determined by immunocytochemistry and Western blot analysis (Figure 3C).

**Figure 4:**
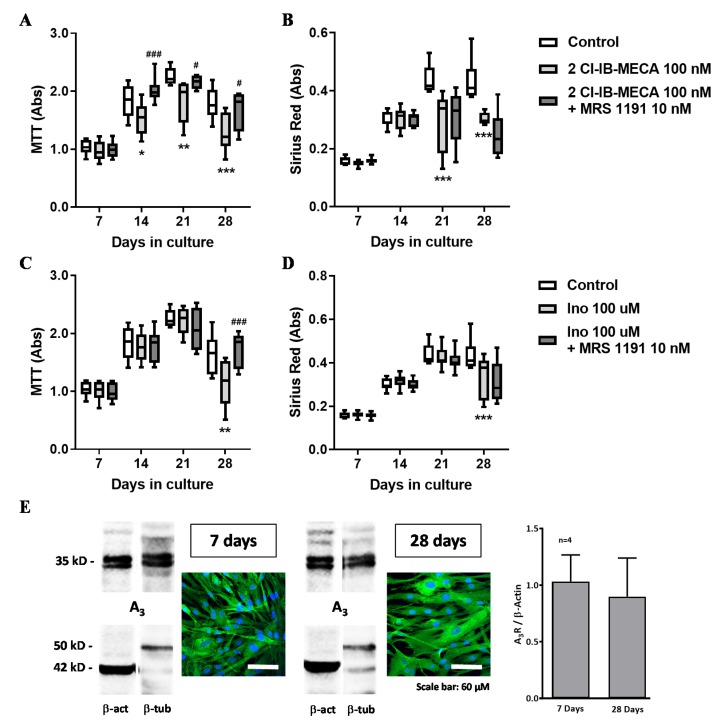
The adenosine metabolite, inosine (100 µM), mimicked the inhibitory effect of the adenosine A_3_ receptor agonist, 2Cl-IB-MECA (100 nM), on cells proliferation/viability (MTT assay, **A** and **C**) and collagen production (Sirius Red staining, **B** and **D**) by human subcutaneous fibroblasts (HSCF) grown in culture for 28 days. Inhibition of HSCF cells proliferation by 2Cl-IB-MECA (100 nM, **A**) and inosine (100 µM, **C**) was attenuated by MRS1191 (10 nM), but the selective A_3_ receptor antagonist did not affect the inhibitory role of 2Cl-IB-MECA (100 nM, **B**) and inosine (100 µM, **D**) on collagen production. Boxes and whiskers represent pooled data from at least three different individuals; 4–6 replicas were performed for each individual. * *p* < 0.05, ** *p* < 0.01 and *** *p* < 0.001 (two-way ANOVA) represent significant differences compared to control conditions; ^#^ *p* < 0.05 and ^###^ *p* < 0.001 (two-way ANOVA) represent significant differences compared to the effect of 2Cl-IB-MECA in the same set of experiments. Panel **E** shows that A_3_ receptor expression (green) was kept fairly constant with time of the cells in culture, as evidenced both by immunofluorescence confocal microscopy and by Western blot analysis normalized either by β-actin (left hand-side blots) or β-tubulin (right hand-side blots) content of the cells. Nuclei are stained in blue with DAPI. Shown are representative data of cells from four different individuals cultured for 7 and 28 days, respectively; experiments were performed in triplicate.

Given that HSCF co-express the adenosine A_3_ receptor throughout the culture period (Figure 4E), but this receptor did not play a role in the pro-fibrotic effect of AMP (Figure 2F), we tested its function on HSCF cell viability/proliferation and collagen production using a highly selective A_3_ receptor agonist, 2Cl-IB-MECA (100 nM). Exposure of HSCF to 2Cl-IB-MECA (100 nM) inhibited cell viability/proliferation (Figure 4A) and collagen production (Figure 4B) at culture days 21 and 28. The anti-proliferative effect of 2Cl-IB-MECA had a higher magnitude while becoming evident from culture day 14 onwards upon increasing the concentration of the A_3_ receptor agonist from 10 to 100 nM (data not shown). Inhibition of HSCF cells growth by 2Cl-IB-MECA (100 nM) was prevented by blocking the A_3_ receptor with MRS1191 (10 nM) (Figure 4A). The action of 2Cl-IB-MECA (100 nM) on collagen production was unaffected by MRS1191 (10 nM) (Figure 4B). Data suggest that, while the adenosine A_3_ receptor exerts a predominant anti-proliferative action on HSCF, adenosine formation from released adenine nucleotides activates preferentially A_2A_ receptors to increase collagen production by these cells.

### 3.5. Inosine Exerts an Anti-Proliferative Role on HSCF through Activation of A_3_ Receptors, but its Formation Requires Exogenous ADA Application

Although inosine was classically considered to be an inactive metabolite of adenosine, activation of adenosine A_3_ receptors by inosine was previously demonstrated [25,26,27,28]. The lack of effect of AMP on HSCF cells growth despite the presence of adenosine A_3_ receptors together with the low expression and activity of ADA in these cells that limit inosine formation from adenosine in these cultures led us to test whether inosine could be the endogenous ligand of A_3_ receptors in HSCF.

Incubation of HSCF with inosine (100 µM) decreased cells viability/proliferation (Figure 4C) and collagen production (Figure 4D) at culture day 28. The inhibitory action of inosine (100 µM) mimicked the effect of the A_3_ receptor agonist, 2Cl-IB-MECA (100 nM) (Figure 4A,B). Likewise, the anti-proliferative effect of inosine (100 µM) was also prevented by MRS1191 (10 nM) (Figure 4C), but the A_3_ receptor antagonist did not affect inhibition of collagen production by inosine (100 µM) (Figure 4D). Results suggest that inosine exerts a predominant inhibitory effect on HSCF cell growth by binding to the adenosine A_3_ receptor.

**Figure 5:**
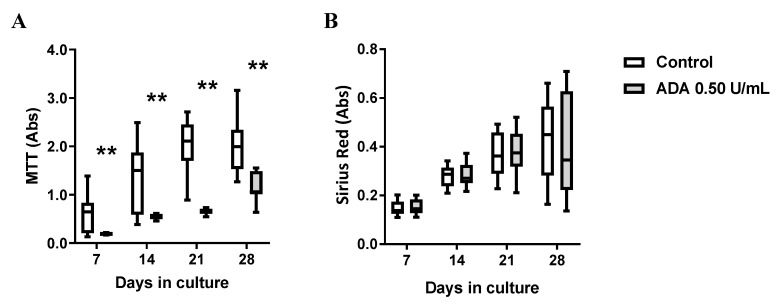
Adenosine deaminase (ADA, 0.5 U/mL) decreases the viability/proliferation (MTT assay, **A**) of human subcutaneous fibroblasts (HSCF) without modifying collagen production (Sirius Red assay, **B**). Boxes and whiskers represent pooled data from at least three different individuals; 4–6 replicas were performed for each individual. ** *p* < 0.01 (one-way ANOVA) represent significant differences compared to control conditions in the same individual.

If our theory is correct, supplementation of culture media with ADA would facilitate adenosine conversion to inosine in HSCF cultures leading to a reduction in cells growth by preferential activation of A_3_ receptors, given that activation of this receptor subtype dependent on sufficient amounts of inosine generated in the cultures. Figure 5 shows that exogenous application of ADA (0.5 U/mL) significantly (*p* < 0.05) decreased HSCF cells viability/proliferation (MTT assay; Figure 5A) without greatly affecting collagen production (Sirius Red assay; Figure 5B) by these cells.

## 4. Discussion

Adenosine is present in most biological fluids. Under basal conditions, the extracellular adenosine concentration is maintained within certain limits (30–300 nM), which are normally above its intracellular concentration because the nucleoside is freely phosphorylated by high affinity intracellular adenosine kinase. Under stressful conditions, extracellular adenosine levels dramatically increase to the micromolar range, allowing the nucleoside to exert multiple actions, namely to protect against ischemic and inflammatory insults as well as to regulate neuronal excitability and the release of neuro- and vasoactive substances [29]. Adenosine most often originates from the extracellular catabolism of adenine nucleotides (danger molecules) released from stressed and/or damaged cells (reviewed in [30]). In previous studies, we demonstrated that fibroblasts of the human subcutaneous tissue respond to inflammatory mediators, such as bradykinin and histamine, by releasing substantial amounts of ATP into the extracellular medium through opening of hemichannels containing connexin-43 and/or pannexin-1 subunits [7,8].

Once released, adenine nucleotides are broken down by NTPDase1 and NTPDase2 bound to the plasma membrane of HSCF [7]. NTPDase1 and NTPDase2 hydrolyze extracellular tri- and diphosphonucleotides with ATP/ADP ratios of ∼1–2:1 and ∼10–40:1, respectively [31]. The kinetic analysis of the extracellular catabolism of ATP and ADP in HSCF indicates that adenine nucleotides were metabolized with a ratio of ∼1.5 (ATP):1(ADP), which is compatible with NTPDase1 being the most effective enzyme in this context [7,8]. The hydrolysis of ATP and ADP by NTPDase1 yielded AMP, which could then be dephosphorylated to adenosine by ecto-5′-nucleotidase/CD73 [32]. The results presented here show that HSCF (as with other mesenchymal-originated cells; see [22,33]) exhibited increasing amounts of the ecto-5′-nucleotidase/CD73 protein with time of the cells in culture. This feature supports a faster conversion of AMP to adenosine than that occurring in many other tissue samples, where ecto-5′-nucleotidase/CD73 is normally the rate limiting enzyme of the ectonucleotidase cascade [34,35,36,37,38,39]. Interestingly, overexpression of subcutaneous NTPDase1 was reported in a model of chronic inflammation [40]. Inflammatory infiltrates, including T lymphocytes endowed with the ecto-5′-nucleotidase/CD73 enzyme, may additionally contribute to fast AMP dephosphorylation and surplus adenosine formation, leading to unpredictable P1 receptor-mediated responses in the inflamed subcutaneous tissue.

**Figure 6:**
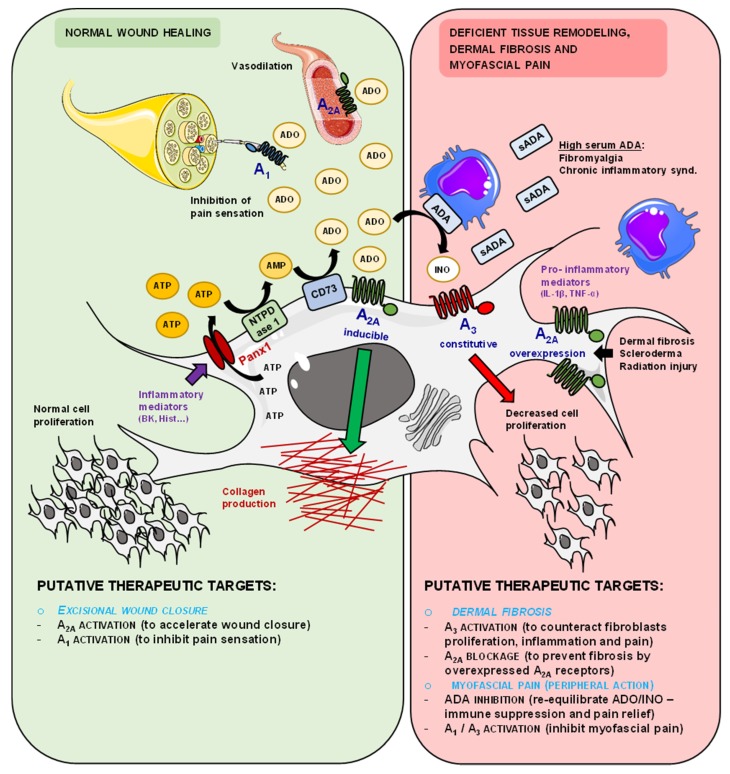
Fine-tuning control of adenosine (ADO) A_2A_ and A_3_ receptors activation in human subcutaneous fibroblasts (HSCF) and its putative pathophysiological implications in wound healing, dermal fibrosis, and myofascial pain. Stressed HSCF release huge amounts of ATP to the extracellular medium, actions of which are rapidly terminated by NTPDase1, resulting in the formation of AMP. HSCFs exhibit high amounts of ecto-5′-nucleotidase/CD73, leading to fast dephosphorylation of AMP into ADO, which tends to accumulate in the extracellular milieu due to a very low ADO deaminase (ADA) activity in these cells. The close proximity between ecto-5′-nucleotidase/CD73 and the A_2A_ receptor and their parallel induction during HSCF cells maturation favor activation of the A_2A_ receptor by ADO generated endogenously from ATP extracellular breakdown. Thus, during normal wound closure, ADO produced by differentiated HSCF may contribute to normal collagen production and vasodilation (via A_2A_ receptors activation) while reducing pain sensation through stimulation of A_1_ inhibitory receptors on peripheral nerve afferents. A different scenario may, however, occur due to unpredicted inosine (INO) formation as a consequence of ADA-bearing inflammatory cell infiltrates as well as in conditions exhibiting high serum ADA levels, such as fibromyalgia and chronic inflammatory states. INO, via constitutively expressed A_3_ receptors, decrease HSCF growth and consequently collagen production. Thus, the novel anti-fibrotic effect of INO together with the anti-inflammatory and the anti-nociceptive properties of highly selective A_3_ receptor agonists undergoing clinical trials may be useful for the treatment of dermal fibrosis and myofascial pain associated with inappropriate subcutaneous tissue remodeling. Considering that overexpression of A_2A_ receptors is a common feature in fibroblast malignancies (e.g., dermal fibrosis, scleroderma, radiation dermal injury), association between A_3_ receptor agonists and A_2A_ receptor antagonists may also be proposed. On the other hand, re-equilibration of ADO/INO concentration ratio using ADA blockers may be helpful to promote immune suppression and nociception relief in myofascial pain conditions with or without A_1_ and/or A_3_ receptor agonists. See text for additional information. Illustration used elements from Servier Medical Art (http://smart.servier.com).

Apart from adenosine formation rate via the ectonucleotidase cascade, the extracellular concentration of the nucleoside near its receptors is strictly regulated by its inactivation pathways, both cellular uptake and ADA. Systemic elevation of adenosine levels in ADA knockout mice has been related to signs of fibrosis in the lungs, the liver, and the kidneys, suggesting a pro-fibrotic trend when ADA is scarce [41]. Moreover, the pharmacological blockade of the A_2A_ receptor prevented dermal fibrosis in this animal model [42]. The enzymatic kinetic experiments performed in this study together with protein detection by immunofluorescence and Western blot analysis show that HSCFs express very little amounts of ADA. Furthermore, the stoichiometry of extracellular adenosine disappearance and formation of its metabolites, inosine and hypoxanthine, was kept fairly constant throughout time, indicating that adenosine cellular uptake by HSCF is negligible. Thus, adenosine formation from released adenine nucleotides may be unbalanced by the lack of nucleoside inactivation mechanisms, ADA and cellular uptake, favoring adenosine accumulation with little inosine (and hypoxanthine) production in the HSCF microenvironment (Figure 6).

The exact role of adenosine and of its metabolites in subcutaneous tissue homeostasis/remodeling is still controversial, but it certainly deserves attention given our findings showing a tendency for adenosine accumulation in HSCF cultures (this study) and its putative clinical involvement in chronic inflammation and myofascial pain [1,2,3]. Evidence suggests that adenosine promotes fibrosis in several organs through different mechanisms associated with distinct patterns of adenosine receptors expression and activation [13,41]. Notwithstanding this, it was also demonstrated that the same adenosine receptor subtype may inhibit or promote fibrosis depending on the target tissue [12]. Adenosine receptors were identified in human dermal fibroblasts, yet, to our knowledge, no previous attempts have been made to characterize the receptor subtypes predominantly involved in the effect of endogenously produced adenosine from adenine nucleotides by human subcutaneous (hypodermal) fibroblasts.

Data from immunofluorescence confocal microscopy show that adenosine A_2A_ and A_3_ receptors are the most expressed P1 receptor subtypes in primary cultures of HSCF (first subculture). Despite this, the adenosine precursor AMP consistently increased collagen production by HSCF via preferential adenosine A_2A_ receptors activation without significantly affecting cells growth. This was suggested because AMP-induced collagen production was mimicked by treatment of HSCF with the selective A_2A_ receptor agonist, CGS21680 (*K_i_* = 27 nM in human cells; [43]), applied in the nanomolar concentration range. Moreover, the pro-fibrotic effects of AMP and CGS21680 were both abrogated in the presence of SCH442416, which specifically antagonizes the A_2A_ receptor, but not upon blocking the A_3_ receptor with MRS1191. Interestingly, increases in collagen production caused by AMP were more evident at culture days 21 and 28, thus coinciding with the highest ecto-5′-nucleotidase/CD73 and A_2A_ receptor protein amounts in HSCF determined by immunocytochemistry and Western blot analysis. These results suggest that the expression of ecto-5′-nucleotidase/CD73 and A_2A_ receptor proteins is induced during HSCF differentiation by a yet unknown molecular mechanism. A tight association between ecto-5′-nucleotidase/CD73 and the A_2A_ receptor was documented by co-immunoprecipitation and proximity ligation assays [44]. Co-localization of these two proteins was also demonstrated by immunofluorescence confocal microscopy in GFAP-positive astrocytes of the human hippocampus [45]. Therefore, ecto-5′-nucleotidase/CD73 is positioned ideally to promote the A_2A_ receptor activation after conversion of AMP into adenosine (Figure 6). Preferential activation of A_2A_ receptors by adenosine generated from the extracellular breakdown of released adenine nucleotides was observed by our group in other locations besides the central nervous system (e.g., neuromuscular junction, myenteric plexus) [35,46].

The A_2A_ receptor seems to be the dominant adenosine receptor in the skin, where it promotes wound healing and excisional wound closure that is normally accompanied by increased collagen (matrix) deposition [12]. This receptor may also play a role in the pathogenesis of fibrotic malignancies of the skin, such as dermal fibrosis. The adenosine A_2A_ receptor is overexpressed in fibroblasts of scleroderma patients presenting excessive collagen deposition in the skin and the visceral organs [47]. Thus, blockade of the adenosine A_2A_ receptor has been proposed to prevent dermal fibrosis and scarring by reducing the collagen content and its misalignment (reviewed in [24,42]) (Figure 6). Our findings showing that adenosine A_2A_ receptors preferentially mediate AMP-induced collagen production by HSCF fully agree with previous assumptions, yet controversy still exists regarding the downstream intracellular pathways responsible for A_2A_ receptor-mediated effects in HSCF. Here, we suggest that AMP applied for up to 28 days favors collagen production by HSCF (with only one passage) through activation of adenosine A_2A_ receptors coupled to the AC/EPAC pathway with no changes being detected upon blocking PKA with H-89. Fine-tuning regulation of type I vs type III collagen production by adenosine A_2A_ receptors activation coupled to the cyclic AMP/PKA/EPAC pathway was reported in human dermal fibroblasts [24]. These authors showed a dual role of the A_2A_ receptor agonist, CGS21680, on type I vs. III collagen production via preferential activation of PKA and EPAC2 when the adenosine analogue was used for periods up to 4 h in the nanomolar and the micromolar concentration ranges, respectively. In our study, we used the Sirius Red assay, which does not distinguish between type I and III collagen production [23]. Nevertheless, one may speculate that the preferential coupling of adenosine A_2A_ receptor activation to the EPAC pathway in HSCF is more likely to increase the proportion of type III over type I collagen, as detected in the granulation tissue and immature scars, where the local concentrations of adenine nucleotides and adenosine are elevated [24,48]. This hypothesis is corroborated by the fact that high intracellular cyclic AMP levels achieved by inhibiting the cyclic AMP specific type IV phosphodiesterase with rolipram mimicked the pro-fibrotic role of A_2A_ receptors activation with CGS21680 or the adenosine precursor, AMP.

Although the adenosine A_3_ receptor subtype was constitutively expressed in fairly constant amounts throughout time, this receptor did not play a role in the pro-fibrotic effect of AMP in HSCF cultures. This prompted us to investigate the activity of the A_3_ receptor in cells viability/proliferation and collagen production using a highly selective A_3_ receptor agonist, 2Cl-IB-MECA. Data show that activation of the A_3_ receptor with 2Cl-IB-MECA decreased cells growth in a MRS1191-sensitive manner, confirming that adenosine A_3_ receptors are fully operative in these cells. Interestingly, exogenously added ADA or the adenosine deamination metabolite, inosine, mimicked the anti-proliferative effect of the A_3_ receptor agonist. These findings show here, for the first time, that AMP, through its breakdown into adenosine, exerts a predominant pro-fibrotic action on HSCF via preferential A_2A_ receptors stimulation, but it may decrease cells growth upon increasing ADA activity, resulting in substantial inosine formation and A_3_ receptors activation under pathological conditions (Figure 6; see discussion below). It is worth noting that inosine is a putative adenosine A_3_ receptor agonist in several preparations [25,26,27,28].

The adenosine A_3_ receptor activation elicits multiple signaling pathways within a cell ending up to the control of many different roles depending on the cell type and agonists concentrations. Upon activation of the A_3_ receptor, (1) the Gα subunit may dissociate from its G_βγ_, decreasing the catalytic activity of adenylyl cyclase and cyclic AMP production, (2) phospholipase C is activated, leading to Ca^2+^ increase or PI3K, Akt phosphorylation, (3) G protein RhoA and phospholipase D are stimulated, (4) mitogen-activated protein kinase (MAPK) family, such as extracellular regulated kinases (ERKs), MAPK-interacting kinase (MNK), and p38, are modulated, and (5) K_ATP_ channels are opened; downstream these pathways, are a series of transcription factors, such as the nuclear factor kappa-light-chain-enhancer of activated B cells (NF-κB), the cAMP response element-binding protein (CREB), the hypoxia-inducible factor 1-alpha (HIF-1α), and the c-myc (reviewed in [18]). Mechanical loading of rat subcutaneous fibroblasts increases adenosine levels and upregulates A_3_ receptors expression and signaling through the MAPK signaling pathway, resulting in modification of cells proliferation and immune-related factors production [49]. Among the MAPK family, which is subdivided into extracellular regulated kinases, such as ERK1/2, and stress-activated protein kinases (SAPK), such as p38 and jun-N-terminal kinase (JNK), the ERK1/2 seems to represent the preferential pathway for cell division and proliferation responses. Therefore, one may speculate that this pathway may act downstream A_3_ receptors activation, yet further studies are required to elucidate whether this mechanism is involved in the anti-proliferative role of inosine in HSCF.

Overall, data enlighten the idea that adenosine, via preferential activation of A_2A_ receptors in fibroblasts, might be a key player in human subcutaneous connective tissue homeostasis and normal wound repair, while excessive scarring and dermal fibrosis may occur when this receptor is overexpressed (Figure 6). For instance, adenosine A_2A_ receptor antagonists have been proposed to prevent radiation-induced dermal injury [50]. Disturbed adenosine homeostasis in the subcutaneous tissue may happen upon infiltration by ADA-bearing inflammatory cells and/or in situations occurring with increases in serum ADA levels, such as fibromyalgia and other chronic inflammatory conditions [51]. Under such circumstances, the unpredicted breakdown of adenosine into inosine, and subsequent activation of A_3_ receptor in HSCF may favor deficient connective tissue remodeling and failure of its viscoelastic properties. Given the fact that subcutaneous connective tissue forms an interconnected net throughout all body [52,53], increases in tissue tension can waveform propagate the release of adenine nucleotides and inosine formation whenever ADA is available, resulting in global changes in the connective tissue network further restraining its compliance. This feature may be the basis for enhanced peripheral inputs and superfluous nociceptive transmission from peripheral soft tissues, which might be involved in the pathogenesis of chronic myofascial pain (Figure 6).

Paradoxically, the adenosine A_3_ receptor was recently implicated in the anti-hyperalgesic effect of adenosine in several chronic pain models [54], mainly through blockage of N-type Ca^2+^ currents and of action potential generation in sensory neurons [55]. Likewise, the role of adenosine in acupuncture-mediated antinociception in human subjects (most probably via A_1_ receptors activation) has been proposed by demonstrating that acupuncture needle rotation transiently increases interstitial adenine nucleotides, whereas adenosine accumulates for more one hour in subcutaneous microdialysates [56]. Under such conditions, adenosine most probably originates from the extracellular breakdown of adenine nucleotides released from subcutaneous fibroblasts given to the fact that (1) remodeling of tissue fibroblasts has been noted in response to mechanical acupuncture [57], and that (2) fibroblasts possess the required machinery (e.g., connexin-43 and/or pannexin-1 hemichannels) to release purines under multiple stressful conditions [7,8]. Thus, we believe that, although the interplay between connective tissue manipulation, inflammation, and remodeling *vis a vis* pronociceptive overactivation is rather complex, purines (in particular adenosine) might play relevant modulatory roles in the pathophysiology of chronic myofascial pain. In this regard, parallel overexpression of A_2A_ receptor and ecto-5′-nucleotidase/CD73 was recently associated with transition from acute to chronic neuroinflammatory conditions [58].

Despite the novelty of the information provided in this study, which was designed to investigate the role of endogenously generated adenosine in primary cultures of HSCF, there are certain limitations deserving investigation in the near future. First, in this study, HSCF cells growth and collagen production were investigated under basal (non-stimulated) conditions; it is expected that the fine-tuning control of HSCF cells function by A_2A_ and A_3_ receptors might change in cells stimulated with TGF-β and pro-inflammatory mediators (e.g., bradykinin, histamine, IL-1β, TNF-α). Second, no animal/tissue model of wound healing, dermal fibrosis, and myofascial pain was presented; discussion regarding these issues was fully based on data from the literature using uneven experimental approaches. Third, besides the use of exogenously added ADA, no attempts have been made thus far to simulate the subcutaneous microenvironment resulting from infiltration by ADA-bearing inflammatory cells in the vicinity of HSCF. Fourth, repercussions of endogenous adenosine production by HSCF on subcutaneous vasculature and afferent nerve fibers also remain to be elucidated in more integrative tissue models before any firm theory can be reached.

In conclusion, data suggest that the HSCF purinome comprising release sites, ectonucleotidases, and purinoceptors deserves to be explored in the future using multitarget drugs and/or monoclonal antibodies, for instance, against ecto-5′-nucleotidase/CD73 activity applied either alone or in combination with subtype selective adenosine receptor antagonists, in order to show their potential for the treatment of inappropriate wound healing, dermal fibrosis, and myofascial pain (see Figure 6).

## Supplementary Materials

The following are available online at https://www.mdpi.com/2073-4409/9/3/651/s1, Figure S1: Positive identification of adenosine A_1_ and A_2B_ receptors on cultured human primary bone marrow stromal cells undergoing osteogenic differentiation.
