# Supplementary material for: Opposing Effects of Adenosine and Inosine in Human Subcutaneous Fibroblasts May Be Regulated by Third Party ADA Cell Providers
Source: Cells. 2020 Mar 7;9(3):651. doi: 10.3390/cells9030651 (PMC7140481; doi:10.3390/cells9030651)
Supplement: Supplementary file 1 [file cells-09-00651-s001.pdf]

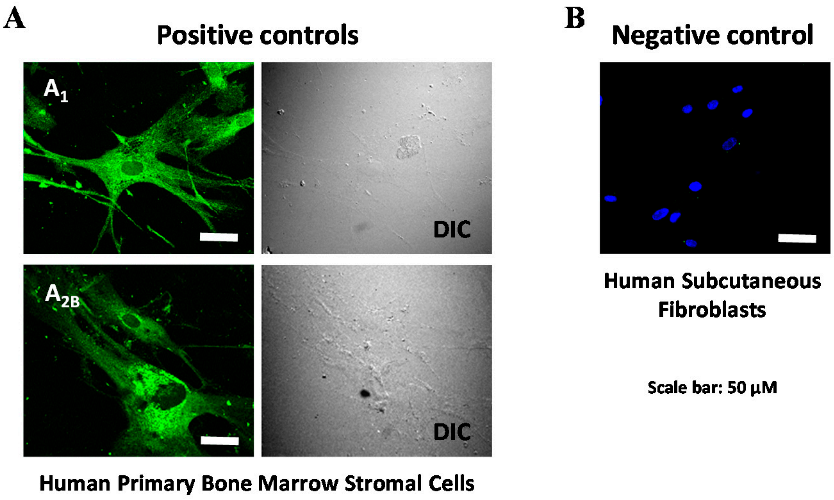

Figure S1. Panel A, shows positive identification of adenosine A_1 and A_{2B} receptors on cultured human primary bone marrow stromal cells undergoing osteogenic differentiation. Cells grown for 21 days in 8-well chamber slides were processed for immunocytochemistry with the same experimental procedure and antibodies (anti-A_1 #AB1587P and anti-A_{2B} #AB1589P from Chemicon, Temecula, CA, USA) used for HSCF (e.g. Figure 1; for details see Materials and Methods). Visualization was performed using the same settings in the confocal microscope (Olympus FV1000, Tokyo, Japan); Differential interference contrast (DIC) images are shown for comparison. Panel B, shown is a negative control carried out by replacing specific primary antibodies by non-immune serum; nuclei are stained with DAPI (blue). Scale bar = 50 µm.
